# New Structure Sheds Light on Selective HIV-1 Genomic RNA Packaging

**DOI:** 10.3390/v7082846

**Published:** 2015-08-24

**Authors:** Erik D. Olson, William A. Cantara, Karin Musier-Forsyth

**Affiliations:** Department of Chemistry and Biochemistry, Center for Retrovirus Research, and Center for RNA Biology, The Ohio State University, Columbus, OH 43210, USA; E-Mails: olson.249@osu.edu (E.D.O.); cantara.2@osu.edu (W.A.C.)

**Keywords:** HIV, RNA structure, retroviral packaging, NMR, psi

## Abstract

Two copies of unspliced human immunodeficiency virus (HIV)-1 genomic RNA (gRNA) are preferentially selected for packaging by the group-specific antigen (Gag) polyprotein into progeny virions as a dimer during the late stages of the viral lifecycle. Elucidating the RNA features responsible for selective recognition of the full-length gRNA in the presence of an abundance of other cellular RNAs and spliced viral RNAs remains an area of intense research. The recent nuclear magnetic resonance (NMR) structure by Keane *et al*. [1] expands upon previous efforts to determine the conformation of the HIV-1 RNA packaging signal. The data support a secondary structure wherein sequences that constitute the major splice donor site are sequestered through base pairing, and a tertiary structure that adopts a tandem 3-way junction motif that exposes the dimerization initiation site and unpaired guanosines for specific recognition by Gag. While it remains to be established whether this structure is conserved in the context of larger RNA constructs or in the dimer, this study serves as the basis for characterizing large RNA structures using novel NMR techniques, and as a major advance toward understanding how the HIV-1 gRNA is selectively packaged.

During the late stage of the human immunodeficiency virus (HIV)-1 life cycle, the major structural and enzymatic proteins of the virus, group-specific antigen (Gag) and GagPol (Pol, polymerase), help to coordinate the assembly of all necessary factors to generate newly infectious virions [2]. These factors include other proteins, as well as several important RNAs [3]. Chief among these selectively-packaged RNAs is a single copy of unspliced, dimeric HIV-1 genomic RNA (gRNA), which serves as the genetic material in progeny virions. gRNA is significantly enriched within virions relative to levels found in the cytoplasm of an infected cell [4,5]. Numerous studies support the notion that the gRNA is recognized by Gag in the cytoplasm [6,7,8], although GagPol has also been proposed to play an important role [9]. Live cell microscopy experiments are consistent with a small number (<10) of Gag molecules selecting gRNA in the cytoplasm prior to assembly at the plasma membrane [10,11,12]. Recently, Keane *et al*. elucidated the nuclear magnetic resonance (NMR) structure of a core HIV-1 RNA element that drives gRNA packaging, yielding significantly new insights into the mechanism by which this process occurs [1].

While many details regarding the process of selective HIV-1 genome incorporation are known, a number of open questions remain [13]. There are at least three fates of HIV-1 viral RNA (vRNA) once it is transcribed in the nucleus [13]: (1) splicing followed by nuclear export and use as a template for translation of HIV-1 regulatory and accessory proteins; (2) export as full-length unspliced vRNA and use as a template for translation of polyproteins Gag and GagPol (Figure 1a); and (3) export as full-length unspliced vRNA that is preferentially selected by Gag for packaging as gRNA (Figure 1b,c). Given that only unspliced, dimeric gRNAs are enriched in virions, mechanisms must exist that allow Gag to differentiate between this form of RNA over cellular RNAs and spliced vRNAs.

Despite nearly two decades of study, the minimal element that confers the selective packaging advantage to gRNA has yet to be unambiguously defined. Elements within the 5′UTR of the HIV-1 genome are clearly necessary (but not sufficient) for conferring specific gRNA packaging. Key elements of the Psi (packaging signal) region include several high-affinity binding sites recognized by the nucleocapsid (NC) domain of Gag: stem loops 1, 2, 3, and 4 (SL1–SL4) (Figure 1) [13,14,15]. This region is located downstream of the reverse transcription primer-binding site and extends just beyond the *gag* start codon. Interestingly, microscopy studies show that viral genomes are not retained at the plasma membrane when their packaging signals were mutated [12] and mutation or deletion of Psi reduces gRNA packaging efficiency by 80%–90% [5,14,16,17]. However, in contrast to other retroviruses such as the Moloney murine leukemia virus (MoMuLV) [18,19,20,21] or Rous sarcoma virus [22,23,24,25] whose minimal Psi elements have been more clearly defined, the identity of this element in HIV-1 is still an active area of investigation. Regions upstream of SL1-SL4 in the 5′UTR and downstream elements in the *gag* gene have been proposed to affect incorporation [26]. More distal elements within the gRNA, such as the GagPol frameshift signal [9] and the Rev response element [27], have also been reported to affect packaging efficiency, although to a lesser extent than those elements found near the 5′ terminus. While the minimal element that is both necessary and sufficient to confer selective HIV-1 gRNA packaging is not yet known, Telesnitsky, Summers and co-workers used a gRNA packaging competition assay to define a “core encapsidation signal” composed of the U5 and Psi (SL1-SL4) regions [28,29]. In this assay, two HIV genome-producing vectors are transfected into cells, one that lacks the 5′UTR entirely (Psi-) and one that contains either wild type or mutant forms of the 5′UTR (test), and the relative levels of the two RNAs that are incorporated into virions are assessed. The authors found that the transactivation response (TAR), polyadenylation signal (polyA), and primer binding site (PBS) regions of the 5′UTR, as well as the start of the *gag* gene were dispensable for maintaining wild-type packaging levels, leaving the Psi and U5 region as the minimal packaging element [29].

**Figure 1:**
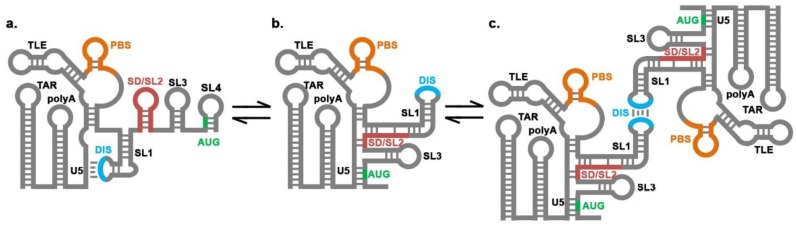
Proposed conformational changes of the HIV-1 5′UTR. (**a**) In the unspliced monomeric RNA, the dimerization initiation site (DIS) sequence participates in a “fold-back” interaction with the U5, thus exposing the *gag* start codon (AUG) for translation; (**b**) The dimerization-competent form of the RNA requires a secondary structure change in which the AUG interacts with the U5 sequence, the major splice donor site/stem-loop 2 (SD/SL2) is sequestered by base pairing with sequences in stem-loop 1 (SL1) and U5, and the DIS is exposed; (**c**) The exposed DIS in the dimerization-competent conformation is then available to form a “kissing”-loop interaction, resulting in a gRNA homodimer that is eventually stabilized into an extended dimer structure. The sequences in orange, cyan, red, and green represent the primer binding site (PBS), DIS, SD/SL2 and the AUG, respectively.

In addition to primary sequence, RNA conformation has also been suggested to play a critical role in selective gRNA incorporation. The HIV-1 5′UTR has been shown to be capable of adopting multiple stable secondary structures [28,30,31], with the conformation favoring gRNA dimerization displaying more favorable NC interaction properties (Figure 1b,c) [28]. The dynamic nature of this region of the genome has resulted in as many as 20 different secondary structure models [14] and has made 3D structure determination extremely challenging. High-resolution structures of individual stem-loop elements SL1-SL4 have been solved [32,33,34,35]. The apo forms of these structures revealed interesting structural motifs and exposed guanosine residues where NC would likely bind specifically. Subsequent studies reported structures of two of these stem loops (SL2 and SL3) in complex with NC [36,37], revealing a guanosine-binding pocket and highly adaptive interactions explaining the high affinity with which NC binds to a variety of RNAs. In addition to possessing high-affinity NC binding sites, SL1 contains the dimerization initiation site (DIS) loop that is critical for gRNA dimerization, and structures have also been solved of the “kissing”-loop [38,39,40,41,42] and extended duplex forms of the SL1 dimer [43,44]. 

While early structures of individual stem-loop elements revealed critical insights into NC-RNA binding, these isolated interactions are not sufficient to explain the highly-efficient process of gRNA packaging. Thus, despite a great deal of work, a structural explanation for why Gag packages only full-length, unspliced gRNA dimers with exquisite selectivity is lacking. Previous efforts to characterize the 3D structure of the multiple stem-loop HIV-1 Psi domain have employed a variety of techniques including mass spectrometry (MS) [45], Förster resonance energy transfer (FRET) [46], and small-angle X-ray scattering (SAXS) [47] to generate lower-resolution structural models of the RNA. In the MS and SAXS studies, ~100-nucleotide constructs of Psi were used; these constructs lacked the nucleotides directly upstream of SL1 to which SL2 is proposed to base pair in the new structure elucidated by Keane *et al*. (see below). In the SAXS structure containing SL1-SL3, the three helices are extended and directed away from one another such that SL1 and SL3 are coaxially stacked and each hairpin is solvent exposed [47]. SL2 is the least well defined of the three helices. Based on the MS study, it was concluded that the domain is more globular with interhelical interactions [45]. The FRET study used a larger ~240 nucleotide RNA including the U5, PBS, and Psi domains and similar to the SAXS study, supported a model in which the helices are solvent-exposed without any long-range interhelical interactions [46]. While useful information was gained from these studies, molecular-level details that may explain selective packaging were needed. The work by Keane *et al*. breaks new ground by elucidating the first high-resolution structure of the HIV-1 core encapsidation signal in the dimerization-competent conformation, revealing a novel topology of the stem loops and a potential explanation as to why this form of gRNA is selectively incorporated by Gag [1].

Other retroviruses have proven to be useful in furthering our understanding of how gRNA is selected by Gag. In the case of MoMuLV, NMR structures have been solved of the minimal Psi (which is composed of three stem loops), both in the apo [48] and NC-bound forms [49]. A crucial observation in these studies was the structural visualization of MoMuLV Psi in two distinct conformations. These structures revealed that high-affinity NC binding sites were only exposed in the dimer conformation, explaining the virus’ preference for dimeric gRNA. Whether the same mechanism holds true for HIV-1 gRNA is unknown.

In the new work, Summers and co-workers determined a high-resolution structure of the 155-nucleotide core encapsidation signal within the HIV-1 5′UTR that can effectively compete with the full-length wild-type gRNA for packaging into virions [1,29]. Importantly, binding features of the viral NC protein and NMR chemical shifts of the core sequence are comparable to those of the full 5′UTR [29]. Solving the structure of this RNA represents a tour de force in NMR structure determination; prior to this work, the largest structure solved by NMR was the 101-nucleotide MoMuLV packaging element also elucidated by Summers and co-workers [48]. Calculating NMR-restrained structures of large RNAs poses significant challenges that must be overcome. Primary among these challenges is the high degree of spectral overlap that occurs due to chemical similarity between the nucleotides. To overcome this, the authors prepared their RNA using differential 2H labeling. Briefly, deuterium is invisible to NMR, therefore combinations of protonated, perdeuterated and deuterated nucleotides produce spectra consisting of resonances corresponding to only the known nonexchangable, nondeuterated protons. To identify signals that could not be assigned, they further utilized a fragmentation method in which the sequence from 105 to 254 was annealed to the sequence from 264 to 345 with the separate strands differently labeled. While the thermodynamics of folding a fragmented RNA can cause significantly different conformations, the authors note that the spectra for the fragmented and parent RNAs were consistent. These strategies yield much simpler spectra with significantly less spectral overlap and can also assist with signal assignments. In addition, truncating the 5′UTR by removing TAR and polyA hairpins, substituting the majority of the PBS domain with a GAGA tetraloop, and substitution of the SL1 loop with a second GAGA sequence minimized spectral overlap and complications associated with gRNA dimerization and conformational heterogeneity.

One of the key findings of this work was that their construct did not exhibit spectra consistent with formation of a SL2 hairpin proximal to the major splice donor site. Indeed, the results suggested an alternative long-range base pairing interaction between “SL2” residues and nucleotides directly upstream of SL1 (Figure 1b). As documented by the authors, this alternative secondary structure is consistent with recently collected selective 2′-hydroxyl acylation analyzed by primer extension (SHAPE) reactivity data [50] and results in sequestration of the splice site. The overall 3D structure of the element indicates a tandem 3-way junction with a tetrahedral-like overall geometry such that there is a clustering of unpaired guanosines near the junctions. The authors hypothesized that these residues may be important for packaging, as these junction structures would be exclusive to the full-length, dimerization-competent gRNA and represent favored NC binding sites [15,28]. Importantly, mutation of the unpaired junction guanosines resulted in significantly weaker binding by NC and reduced packaging efficiency relative to the wild-type RNA, supporting the authors’ hypothesis. The elucidation of this novel structure suggests a mechanism for how Gag discriminates against spliced vRNAs. Given that SL2 contains the most used 5′ splice donor site, all spliced vRNAs contain a truncated SL2 and lack SL3-SL4 [51]. This would compromise the ability of the 5′UTRs from these spliced transcripts to form the tandem 3-helix junction motif required to expose the cluster of guanosines critical for packaging.

A previous finding by the same group showed that the loop residues of SL1 exhibit a long-range “fold-back” interaction with U5, allowing exposure of the *gag* start codon in the monomeric form of the gRNA (Figure 1a) [28]. The U5:SL1 interaction is not possible in the dimerization-competent structure in their current work, further supporting the notion that the monomeric (Figure 1a) and dimerization-competent (Figure 1b) structures are mutually exclusive, with only the latter harboring the tandem 3-way junction and clustering of unpaired guanosines that promote selective gRNA packaging. Therefore, the monomeric gRNA conformation would presumably not be as efficiently packaged as the dimer; however, this has yet to be directly tested. Taken together with the earlier work, these data predict a large conformational switch between the monomeric and packaging competent dimeric forms of the RNA, requiring a significant secondary and tertiary structure rearrangement.

The recent work by Keane *et al*. is an important step forward in many respects. From a technological point of view, the authors demonstrate the capability of NMR to determine 3D structures of large RNAs. In addition to the technological breakthrough, this work makes a significant contribution toward understanding how HIV-1 can efficiently package the correct form of its genetic material. The finding that the HIV-1 Psi stem loops in the dimerization-competent conformation adopt a distinct topology containing a tandem 3-helix junction, thus exposing clusters of unpaired guanosines, may help explain Gag/NC’s preference for this form of gRNA. This exposure of critical guanosine residues upon dimerization is reminiscent of the mechanism MoMuLV uses to ensure only the dimeric form of its genome is packaged [48,49,52]. It is tempting to hypothesize that retroviruses may have maintained a conserved mechanism to ensure selective packaging of their gRNA.

Despite the significant insights gained into this important biological question, many questions remain unanswered. For example, a high-resolution structure of the monomeric fold-back conformation of the 5′UTR has not yet been determined (Figure 1a), and at least 3 different long-range interactions have been proposed [28,30,31]. The factor or factors that trigger the proposed conformational switch also remain unknown. This change may be triggered by tRNA primer annealing to the PBS [31], Gag binding and chaperone activity [15,53,54,55,56], and/or the binding of an as of yet unidentified viral or host protein.

This work is an important step forward in clarifying our understanding of the HIV-1 gRNA packaging mechanism; however, some caveats to this work require continued study of this important process. Specifically, although the SHAPE data are consistent with their new secondary structure, they are not exclusive to this structure and were previously used to support a different one [50]. Another caveat of the new NMR study is that it was conducted using a truncation construct lacking the apical 85 nucleotides of the PBS domain. It is unclear how this truncation would affect the final structure of the RNA. It is also not clear whether including the other domains of the HIV-1 5′UTR (*i.e*., TAR and polyA) would alter the overall structure of the core encapsidation signal, and if so, how. Appending large polyanionic domains such as PBS and TAR/polyA may lead to additional long-range interactions or result in a change in the overall shape and/or orientation of the 5′UTR helices. Finally, binding studies were performed with NC alone, while in the physiological context, Gag would be involved in these interactions. Indeed, the binding studies focused on mutagenesis of single-stranded guanosines; however, many of the mutated residues are also single-stranded in previously proposed structures of the 5′UTR. Thus, the mutagenesis results do not exclusively support the newly proposed structure.

In summary, the NMR structure by Keane *et al*. allows a plausible mechanism for genome selection to be proposed. However, additional structural studies using techniques such as SAXS and cryo-electron microscopy, in addition to novel functional investigations, will be needed to validate and extend this model.
